# Exposure Therapy in Mixed Reality for Obsessive-Compulsive Disorder: A Randomized Clinical Trial

**DOI:** 10.1001/jamanetworkopen.2025.11488

**Published:** 2025-05-20

**Authors:** Franziska Miegel, Lena Jelinek, Luzie Lohse, Steffen Moritz, Jannik Blömer, Kim Juckoff, Amir Yassari, Lara Rolvien

**Affiliations:** 1 Department of Psychiatry and Psychotherapy, University Medical Center Hamburg-Eppendorf, Hamburg, Germany

## Abstract

**Importance:**

Exposure therapy in virtual reality (VR) is well established for anxiety disorders. While early studies show promise for VR-based exposure and response prevention (ERP) in obsessive-compulsive disorder (OCD), a key challenge of traditional VR is the weak sense of presence, deemed crucial for effectiveness.

**Objective:**

To evaluate the feasibility and efficacy of ERP in mixed reality (MERP).

**Design, Setting, and Participants:**

This randomized clinical trial (RCT) included 2 outpatient treatment groups, MERP vs self-guided ERP (SERP) from March 15, 2022, to October 26, 2024. Participants were required to be 18 to 80 years of age and have contamination-related OCD (C-OCD). Exclusion criteria consisted of schizophrenia or bipolar disorder, severe substance use disorder, acute suicidality, and ongoing inpatient treatment. Patients’ symptom severity was assessed by the Yale-Brown Obsessive Compulsive Scale (Y-BOCS) before (baseline) and after the 6-week intervention period as well as 3 months after posttreatment (follow-up).

**Intervention:**

Six sessions of MERP or SERP.

**Main Outcomes and Measures:**

Main outcome was the severity of OCD symptoms in the past 7 days on the Y-BOCS. Sense of presence and subjective appraisal were the secondary outcomes.

**Results:**

Thirty-six participants fulfilled inclusion criteria. Twenty-four participants (66.7%) were female; mean (SD) age was 35.42 (14.03) years. Both groups had severe OCD symptoms, with major depressive disorder as the most common comorbidity (23 [63.9%]). Twenty-nine participants (80.6%) completed the postintervention and follow-up assessments. At baseline, patients in the MERP group had a mean (SD) Y-BOCS score of 26.94 (5.83), while the SERP group had a mean (SD) score of 24.22 (4.12). The decline in patients’ OCD symptoms (Y-BOCS) was similar in the MERP (mean difference, 3.15) and the SERP groups (mean difference, 1.47) (η_p_^2^ = 0.002; 95% CI, –3.187 to 3.893). Significant improvements were observed within the MERP group from baseline to post treatment across several psychopathological variables, including OCD symptoms, with a medium to large effect (Cohen *d*, 0.584-0.931; 95% CI, 0.026-1.551). Sense of presence was moderate (mean [SD], ≤3.24 [≤2.07]). Subjective appraisal was heterogeneous.

**Conclusions:**

This RCT on MERP in C-OCD provided important insights. While there were no significant between-group differences, changes in psychopathology within the MERP group suggest some level of effectiveness. However, the limited impact indicates that the full potential of MERP has not yet been realized, and future research should aim to improve immersion and overall effectiveness.

**Trial Registration:**

German Clinical Trial Registry: DRKS00020969

## Key Points

**Question:**

Can mixed reality exposure and response prevention therapy (MERP) effectively reduce symptoms in patients with contamination-related obsessive-compulsive disorder (OCD)?

**Findings:**

In this single-center randomized clinical trial including 36 patients, MERP did not significantly reduce OCD symptom severity compared with an active control group, but qualitative feedback was valuable for revisions.

**Meaning:**

These findings provide insights into MERP’s potential revisions and application in OCD.

## Teaser

This randomized clinical trial compares the efficacy of exposure and response prevention therapy in mixed reality with self-guided exposure and response prevention therapy among patients with contamination-related obsessive-compulsive disorder.

## Introduction

Obsessive-compulsive disorder (OCD) is a disorder with a lifetime worldwide prevalence of 1% to 3%.^1^ Treatment guidelines^2^ recommend cognitive behavioral therapy (CBT) with exposure and response prevention (ERP) as the first-line treatment. In ERP, individuals face OCD-provoking stimuli but are prevented from enacting compulsions. This evidence-based therapeutic approach has exhibited high effectiveness (Hedges *g* = 0.74)^3^ in reducing symptom severity and improving overall functioning. Although CBT with ERP is the most effective treatment for OCD, it is not—or not adequately—used in clinical practice.^4^ Only one-third of therapists apply exposure in vivo,^4^ with just one-third of those adhering to protocols,^4^ highlighting the disparity between recommendations and clinical practice. Senter et al^5^ found that although CBT is recommended to 59% of patients with OCD, only 44% undergo treatment. Barriers to CBT with ERP can be patient related (eg, patient barriers [dropout rate 18.7%]^6^), circumstance based (eg, during the COVID-19 pandemic ERP was used less often due to risk of infection^7^), or therapist related (eg, time-consuming, fear of severe reactions^4^). These challenges, compounded by the difficulty of simulating OCD-relevant situations in clinical settings, contribute to the treatment gap.^4^

Exposure therapy in virtual reality (VR) enables efficient exposure treatment within the therapist’s workspace, eliminating travel time^8^ and ensuring consistent experimental conditions, with extensive research showing comparable or slightly superior effects compared with traditional in vivo exposure for most anxiety disorders.^9,10,11^ It also facilitates standardized therapist-led ERP within a controlled clinical setting, potentially increasing adherence to evidence-based treatment, and it enables the collection of objective data, such as eye-tracking measures, to enhance research, optimize ERP protocols, and improve treatment outcomes. Limited research has focused on applying ERP in VR (VERP) for OCD, and the heterogeneity of symptoms (eg, contamination, sexual obsessions) hinders the generalizability of outcomes. However, studies indicate that it is possible to induce emotional responses (heightened anxiety, increased compulsions) using VERP for patients with OCD.^12^ VERP is feasible and shows potential treatment efficacy.^13^ However, sense of presence is a major issue in studies on VERP. Also, creating new environments for unavailable OCD-provoking stimuli is technically complex.

While VR fully immerses patients into generated environments, augmented reality (AR) glasses superimpose virtual objects onto real surroundings, maintaining visibility and naturalistic movement and thus increasing how real the experience feels to the patient. Sense of presence is especially vital for inducing anxiety and disgust in ERP, and studies have confirmed that AR can induce these emotions in patients with contamination-related OCD (C-OCD).^14^ Mixed reality (MR) goes beyond AR by allowing for real-time interaction and integration between the virtual and physical elements, creating a more immersive and interactive experience. This advancement may further enhance the sense of presence, which is crucial for exposure therapy. However, this must be explicitly examined in future studies using a comparative design among MR, AR, and VR.

While MR is already used in medicine, in psychological treatment it shows promise for ERP-based therapies by offering personalized sessions. Unlike VR, where whole virtual environments must be developed, MR only requires the development of new singular stimuli, which is of benefit for OCD due to its highly idiosyncratic nature. The increased sense of presence in MR fosters deeper engagement,^15^ which especially aids patients with C-OCD, who often require touch to elicit disgust during exposure. A pilot study by some authors of the present study^16^ was the first to date to explore the feasibility of MERP in a clinical sample. Patients with C-OCD were randomized to receive an add-on MERP intervention (6 sessions; intervention group) or continue their inpatient treatment (control group). Safety and acceptance were generally high, indicating the feasibility of MERP. However, participants criticized the realism of the objects and sounds. Based on this feedback, software modifications were made to improve the sense of presence and potentially the effectiveness of MERP.

The present randomized clinical trial (RCT) is the first, to our knowledge, that aims to investigate the efficacy of MERP by comparing it with an active control group (ie, self-guided ERP [SERP]) in outpatients with C-OCD. We hypothesized that the decline in OCD symptoms would be greater in the MERP group than the SERP group from baseline to posttreatment assessment and from baseline to follow-up assessment. We also assumed positive expectations regarding MERP, high acceptance rates, and high ratings of immersion.

## Methods

### Design

This RCT was conducted from March 15, 2022, to October 26, 2024, and examined the efficacy of MERP vs an active control group using SERP. Assessments were conducted at baseline, 6 weeks post treatment, and 3-month follow-up by blinded raters. Participants were randomly assigned to MERP or SERP (1:1) using online software after the baseline assessment by the principal investigators (F.M. and L.R.). Instances of rater unblinding were recorded. All participants provided written informed consent and received €50 for completing all assessments. Standard care was allowed in both groups. The study received ethical approval by our local ethics committee of the Center for Psychosocial Medicine of the University Medical Center Hamburg-Eppendorf, Hamburg, Germany, was registered, and followed the tenets of the Declaration of Helsinki.^17^ The study protocol has been published previously^18^ and the version submitted to the local ethics committee is available in Supplement 1. The study follows the Consolidated Standards of Reporting Trials (CONSORT) reporting guideline.

### Participants and Procedure

Participants were recruited from 2022 to 2023 from a patient database and through various advertisements (eg, newspapers, OCD support groups, social media). Eligibility was screened via telephone, with baseline and posttreatment assessments conducted in person and follow-up conducted via telephone; questionnaires were completed online. The Mini International Neuropsychiatric Interview (German version 7.0.2)^19^ confirmed OCD diagnosis, identified comorbidities, and checked exclusion criteria. Exclusion criteria consisted of past or present diagnosis of schizophrenia or bipolar disorder, current severe substance use disorder, acute suicidality, severe neurological disorders related to OCD, and ongoing inpatient treatment. Participants aged 18 to 80 years with C-OCD, sufficient German language skills, and informed consent were eligible.

### Intervention

#### MERP

**Figure 1.**
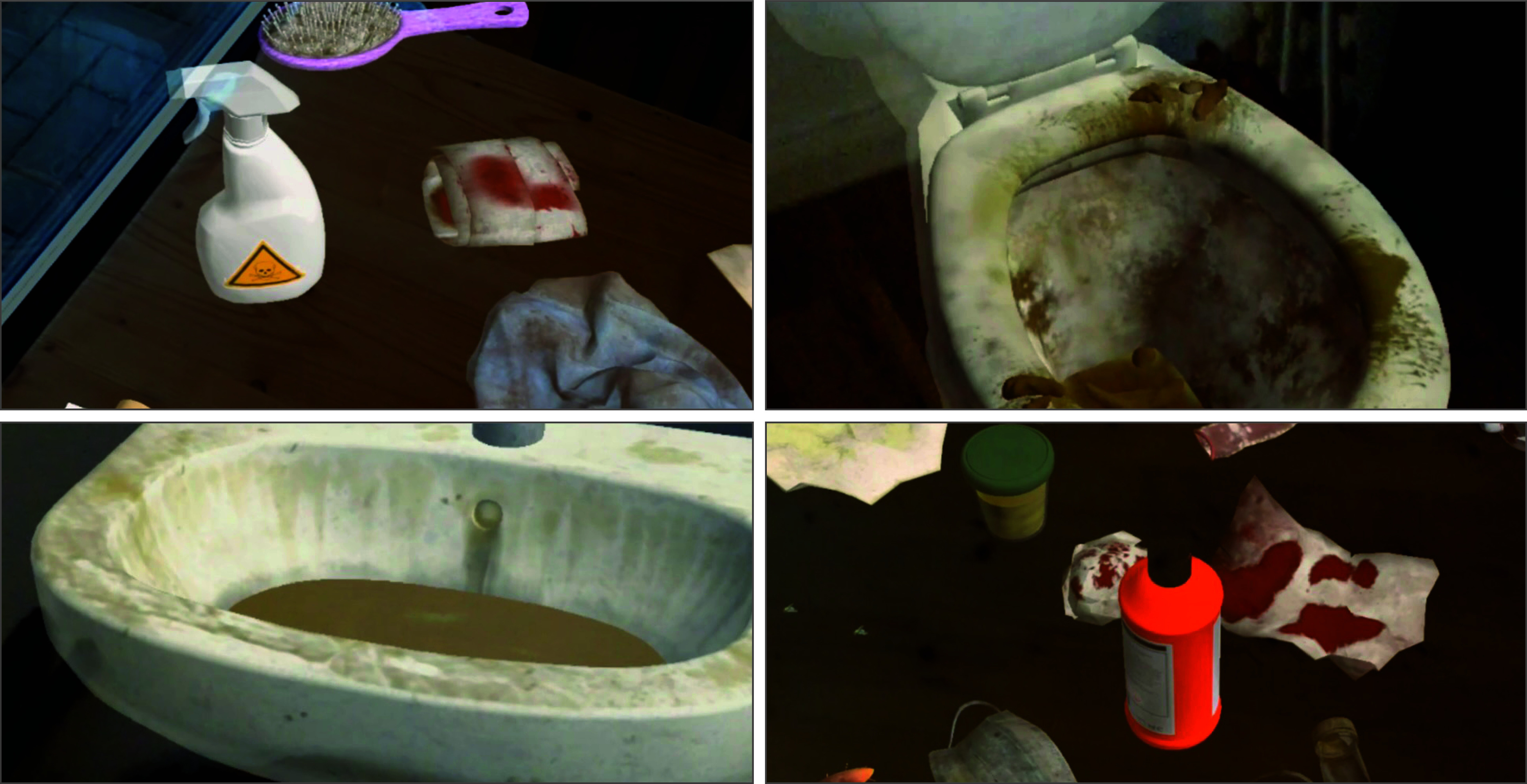
Snapshots of Various Virtual Objects That Were Integrated Into the Real Environment During Mixed Reality Exposure and Response Prevention Unity software version 2020.3.22f1 (Unity Technologies) was used with permission to create the virtual objects within the mixed reality. The authors take responsibility for the content generated.

MERP sessions lasted 60 to 90 minutes and were conducted once a week for 6 weeks by a trained psychotherapist (among others by L.L.) with at least a master’s degree in psychology. In the first session, participants’ medical history was assessed. The second session focused on psychoeducation and preparing for MERP, where participants rated the difficulty of virtual objects and selected those for increasing difficulty from sessions 3 to 6. MERP used MR glasses (Magic Leap One; Magic Leap Inc) with Unity software, version 2020.3.22f1 (Unity Technologies) to scan the real environment and accurately place virtual objects. The therapist used a controller to select and position objects in the room, and while in patient mode, the objects were locked in place for exposure, allowing the patient to interact with them using the controller (Figure 1).

Using the tablet, the therapist could administer an artificial virus scan (green spots marking objects or places of high contamination) and activate coughing sounds in 3 levels of intensity. If the patient touched a previously contaminated object (the virus was represented by green spots), the virus particles spread onto the participant’s hands (eFigure 1 in Supplement 2).

#### SERP

The active control group conducted SERP using a workbook that followed the same structure and content as the therapists’ MERP manual. This included psychoeducation on OCD and ERP, guidance on planning ERPs, and space for documenting experiences. Participants were instructed to complete 1 self-guided ERP exercise per week, for a total of 4 exercises, mirroring the frequency of ERP in the MERP condition. Unlike MERP, participants in SERP selected real objects for their ERPs and performed the exercises independently, without therapist involvement. They were encouraged to document their preparation and progress in the workbook.

### Measures

The Yale-Brown Obsessive Compulsive Scale (Y-BOCS)^20^ was used to assess the severity of OCD symptoms in the past 7 days through a structured interview and served as the main outcome (scores range from 0-40, with higher scores indicating higher OCD symptom severity). The following questionnaires assessed secondary outcome variables: Obsessive Compulsive Inventory–Revised,^21^ Beck Depression Inventory-II,^22^ the 7-item Generalized Anxiety Disorder Scale (GAD-7),^23^ the global item of the World Health Organization Quality of Life Instrument,^24^ treatment expectations, Temple Presence Inventory (only 15 items and their factors were assessed based on ethical considerations and relevance to our research question),^25^ and Subjective Appraisal Rating (based on the scale used by Miegel et al^16^).

### Statistical Analysis

All analyses were conducted with SPSS, version 29.0.1.0 (IBM Corporation). To evaluate the changes in symptoms between the MERP and SERP groups, we used analyses of covariance. Treatment type (MERP vs SERP) served as the between-participant factor, while the differences in outcome scores from baseline to post treatment and baseline to follow-up were the dependent variables. Baseline scores of the respective outcome variable were used as covariates. Paired *t* tests were planned^18^ to assess within-group differences from baseline to posttreatment and follow-up assessments. Complete case (ie, participants who completed all assessments) and intention-to-treat (ITT using the multiple imputation method; participants who completed baseline assessment) analyses were conducted. A 2-sided significance threshold was applied. Sense of presence, expectations, and acceptability of MERP were analyzed descriptively. Exploratory moderation analyses using the Hayes PROCESS macro for SPSS and a process-based regression were conducted with all baseline variables as potential moderators for the change in OCD symptoms (difference score of the Y-BOCS total score from baseline to posttreatment assessment).

The power analysis was conducted with G*Power, version 3.1.9.6.^26^ Based on the results of studies on VR and augmented reality exposure in anxiety disorders and the large effect sizes of 0.90 (Hedges *g* = 0.90^9^; Hedges *g* = 0.33^27^), we anticipated a larger effect size for MERP than for SERP due to an expected higher immersion resulting from the implementation in MR. Based on these assumptions, a sample size of 64 participants was needed to detect a large effect (*f* = 0.4), with a power of .80 and an α level of .05.

## Results

### Sample

**Figure 2.**
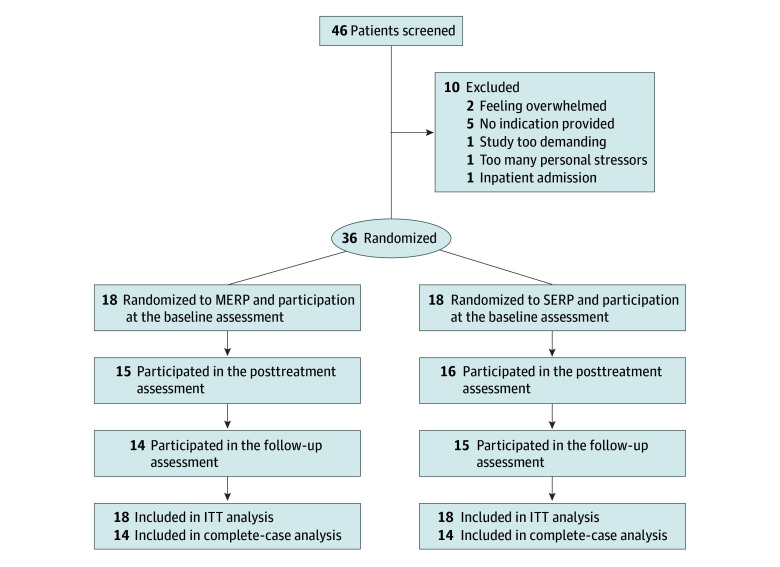
Participant Flow Diagram ITT indicates intention to treat; MERP, mixed reality exposure and response prevention; and SERP, self-guided exposure and response prevention.

Thirty-six participants (18 in the MERP group and 18 in the SERP group) fulfilled inclusion criteria and were included in the final sample (Figure 2). Twenty-four participants (66.7%) were female, 11 (30.6%) were male, and 1 (2.8%) identified as gender diverse; mean (SD) age was 35.42 (14.03) years. As preregistered, we initially planned to recruit a larger sample of 64 participants, but we had to stop after 1.5 years of recruitment due to limited resources. We were unable to reach the targeted sample size because of the restriction to the symptom domain of C-OCD, regional limitations (only patients living nearby could be included, as video-conference treatment was not possible), restrictions during the COVID-19 pandemic, and patients’ fears of coming to the hospital during the pandemic.

The OCD symptoms of both groups could be classified as severe (Table 1). At baseline, patients in the MERP group had a mean (SD) Y-BOCS score of 26.94 (5.83), while the SERP group had a mean (SD) score of 24.22 (4.12). The most prevalent comorbid diagnosis was major depressive disorder (23 [63.9%]; present episode, 3 [8.3%]; past episode, 20 [55.6%]). On average, patients in the MERP group attended 5 sessions (mean [SD], 4.53 [1.77]), with a range from 0 to 6 sessions. Only 1 of 17 participants attended 0 sessions (5.9%) and 6 attended all sessions (35.3%). Most participants were taking psychiatric medication for a mean (SD) duration of 156.50 (259.12) days.

**Table 1.** Sociodemographic and Psychopathological Characteristics at Baseline

Variable	Total sample (N = 36)	MERP (n = 18)	SERP (n = 18)
Sociodemographic			
Gender, No. (%)			
Female	24 (66.7)	13 (72.2)	11 (61.1)
Male	11 (30.6)	4 (22.2)	7 (38.9)
Diverse	1 (2.8)	1 (5.6)	0
Age, mean (SD), y	35.42 (14.03)	32.28 (11.13)	38.56 (16.14)
Formal education, mean (SD), y	11.83 (1.36)	11.94 (1.29)	11.72 (1.45)
Medication used, No. (%)			
Antidepressant	19 (52.8)	10 (55.6)	9 (50.0)
Antipsychotic	0	0	0
Combination	5 (13.9)	3 (16.7)	2 (11.1)
Other	1 (2.8)	1 (5.6)	0
None	11 (30.6)	4 (22.2)	7 (38.9)
Psychopathology scores, mean (SD)			
Y-BOCS total^a^	25.58 (5.16)	26.94 (5.83)	24.22 (4.12)
Y-BOCS obsessions^b^	12.36 (2.84)	13.06 (3.30)	11.67 (2.17)
Y-BOCS compulsions^b^	13.22 (2.78)	13.89 (2.93)	12.56 (2.53)
OCI-R^c^	29.41 (12.81)	32.67 (12.88)	26.17 (12.22)
BDI-II^d^	18.72 (12.21)	21.67 (13.36)	15.78 (10.49)
GAD-7^e^	11.50 (5.84)	12.28 (5.21)	10.72 (6.47)
WHOQOL-BREF global item^f^	2.86 (1.07)	2.78 (0.94)	2.94 (1.21)

Abbreviations: BDI-II, Beck Depression Inventory-II; GAD-7, Generalized Anxiety Disorder Scale-7; MERP, mixed reality exposure and response prevention; OCI-R, Obsessive Compulsive Inventory–Revised; SERP, self-guided exposure and response prevention; WHOQOL-BREF, global item of the World Health Organization Quality of Life Instrument, Short Form; Y-BOCS, Yale-Brown Obsessive Compulsive Scale.

^a^ Scores range from 0 to 40, with higher scores indicating greater symptom severity.

^b^ Scores range from 0 to 20, with higher scores indicating greater symptom severity.

^c^ Scores range from 0 to 72, with higher scores indicating greater symptom severity.

^d^ Scores range from 0 to 63, with higher scores indicating greater symptom severity.

^e^ Scores range from 0 to 21, with higher scores indicating greater symptom severity.

^f^ Scores range from 1 to 5, with higher scores indicating higher quality of life.

### Completion Rate

Twenty-nine participants (80.6%) completed the postintervention and follow-up assessments. Figure 1 presents the flow diagram. The completion rate did not differ between groups (χ^2^_1_ = 0.177; *P* = .67). Those who did and did not complete the assessments did not differ on sociodemographic variables.

### Complete Case and ITT Analyses

For all outcome variables, no significant differences between the MERP group and the SERP group from baseline to posttreatment assessment or from baseline to follow-up in the complete case analysis as well as the ITT analyses were found (Table 2). Dependent *t* tests showed several significant changes in the MERP group (eg, Y-BOCS, *t*_14_ = 2.263; *P* = .02; Cohen *d* = 0.584) (Table 2). One patient in the SERP and 1 in the MERP group responded (reduction in Y-BOCS total score of at least 35%) at posttreatment assessment and 1 patient in the MERP group responded at follow-up assessment. No patient achieved remission (Y-BOCS total score of ≤12) at posttreatment assessment or follow-up.

**Table 2.** Between-Group Differences Across Time for the Primary and Secondary Outcomes

Measure	Baseline score		Posttreatment score		Statistics for complete case sample		*P* value for complete case sample	*P* value for ITT sample	Follow-up score		Statistics for complete case sample		*P* value for complete case sample	*P* value for ITT sample
	MERP group (n = 18)	SERP group (n = 18)	MERP group (n = 15)	SERP group (n = 16)	*F*_1,28_	*η_p_^2^*			MERP group (n = 14)	SERP group (n = 15)	*F*_1,28_	*η_p_^2^*		
Y-BOCS total^a^	27.86 (6.04)	23.40 (4.00)	24.71 (5.62)^b^^,^^c^	21.93 (4.30)	0.042	0.002	.84	.96	25.00 (7.60)^b^^,^^d^	20.60 (4.34)^b^^,^^e^	0.034	0.001	.85	.92
Y-BOCS obsessions^f^	13.71 (3.38)	11.40 (2.20)	12.57 (3.18)	10.47 (2.36)	0.833	0.031	.37	.54	11.86 (3.86)^g^^,^^h^	10.53 (2.61)	0.417	0.016	.52	.45
Y-BOCS compulsions^f^	14.14 (3.06)	12.00 (2.27)	12.14 (2.86)^b^^,^^i^	11.47 (2.32)	0.057	0.002	.81	.66	13.14 (3.84)	10.07 (2.31)^g^^,^^j^	2.017	0.074	.16	.22
OCI-R^k^	31.50 (14.10)	27.00 (11.37)	31.57 (13.70)	29.47 (13.06)	0.158	0.006	.70	.75	32.07 (14.68)	26.40 (9.96)	0.579	0.022	.45	.36
BDI-II^l^	19.21 (13.90)	14.93 (10.37)	17.14 (16.44)^b^^,^^m^	12.87 (11.50)	0.010	0.0004	.92	.67	19.21 (16.05)	15.07 (10.98)	0.001	0.0004	.98	.96
GAD-7^n^	12.21 (5.78)	10.53 (6.69)	10.64 (5.29)^o^^,^^p^	10.33 (5.83)	0.748	0.028	.40	.42	13.00 (5.72)	9.13 (5.17)	3.513	0.119	.07	.08
WHOQOL-BREF global item^q^	2.86 (0.86)	3.07 (1.28)	3.07 (1.07)	2.87 (0.99)	1.468	0.053	.24	.29	2.79 (1.05)	3.07 (1.28)	0.183	0.007	.67	.75

Abbreviations: BDI-II, Beck Depression Inventory-II; GAD-7, Generalized Anxiety Disorder Scale-7; ITT, intention to treat; MERP, mixed reality exposure and response prevention; OCI-R, Obsessive Compulsive Inventory–Revised; SERP, self-guided exposure and response prevention; WHOQOL-BREF, global item of the World Health Organization Quality of Life Instrument, Short Form; Y-BOCS, Yale-Brown Obsessive Compulsive Scale.

^a^ Scores range from 0 to 40, with higher scores indicating greater symptom severity.

^b^ Significant effect at a medium effect size (Cohen *d*>0.05).

^c^ Cohen *d* = 0.58.

^d^ Cohen *d* = 0.66.

^e^ Cohen *d* = 0.77.

^f^ Scores range from 0 to 20, with higher scores indicating greater symptom severity.

^g^ Significant effect at a large effect size (Cohen *d*>0.08).

^h^ Cohen *d* = 0.93.

^i^ Cohen *d* = 0.72.

^j^ Cohen *d* = 0.94.

^k^ Scores range from 0 to 72, with higher scores indicating greater symptom severity.

^l^ Scores range from 0 to 63, with higher scores indicating greater symptom severity.

^m^ Cohen *d* = 0.54.

^n^ Scores range from 0 to 21, with higher scores indicating greater symptom severity.

^o^ Significant effect at a small effect size (Cohen *d*>0.02).

^p^ Cohen *d* = 0.49.

^q^ Scores range from 1 to 5, with higher scores indicating higher quality of life.

### Sense of Presence

The sense of presence was measured by the 3 scales of the Temple Presence Inventory, for which means and SDs were computed. Engagement (mean [SD], 3.24 [2.07]), perceptual realism (mean [SD], 1.97 [1.33]), and spatial presence (mean [SD], 2.74 [1.91]) were all below the midpoint of 4.

### Patients’ Expectations of MERP

Patients’ expectations of MERP were mixed, with all 36 patients (100%) indicating that they thought they would attend all sessions. However, less than half of the participants (17 [47.2%]) expected that MERP would help them to implement what they learned in their everyday life. Half of participants (18 [50.0%]) agreed on the item “I am more likely to learn coping skills in ERP in vivo than in MERP.” Concerns such as dizziness were minimal (5 [13.9%]). In total, 30 participants (83.3%) expressed positive expectations for OCD symptom improvements and 34 (94.4%) found it easier to confront their OCD in the MR environment than in real life. eFigure 2 in Supplement 2 shows all items and answers.

### Subjective Appraisal of MERP and SERP

**Figure 3.**
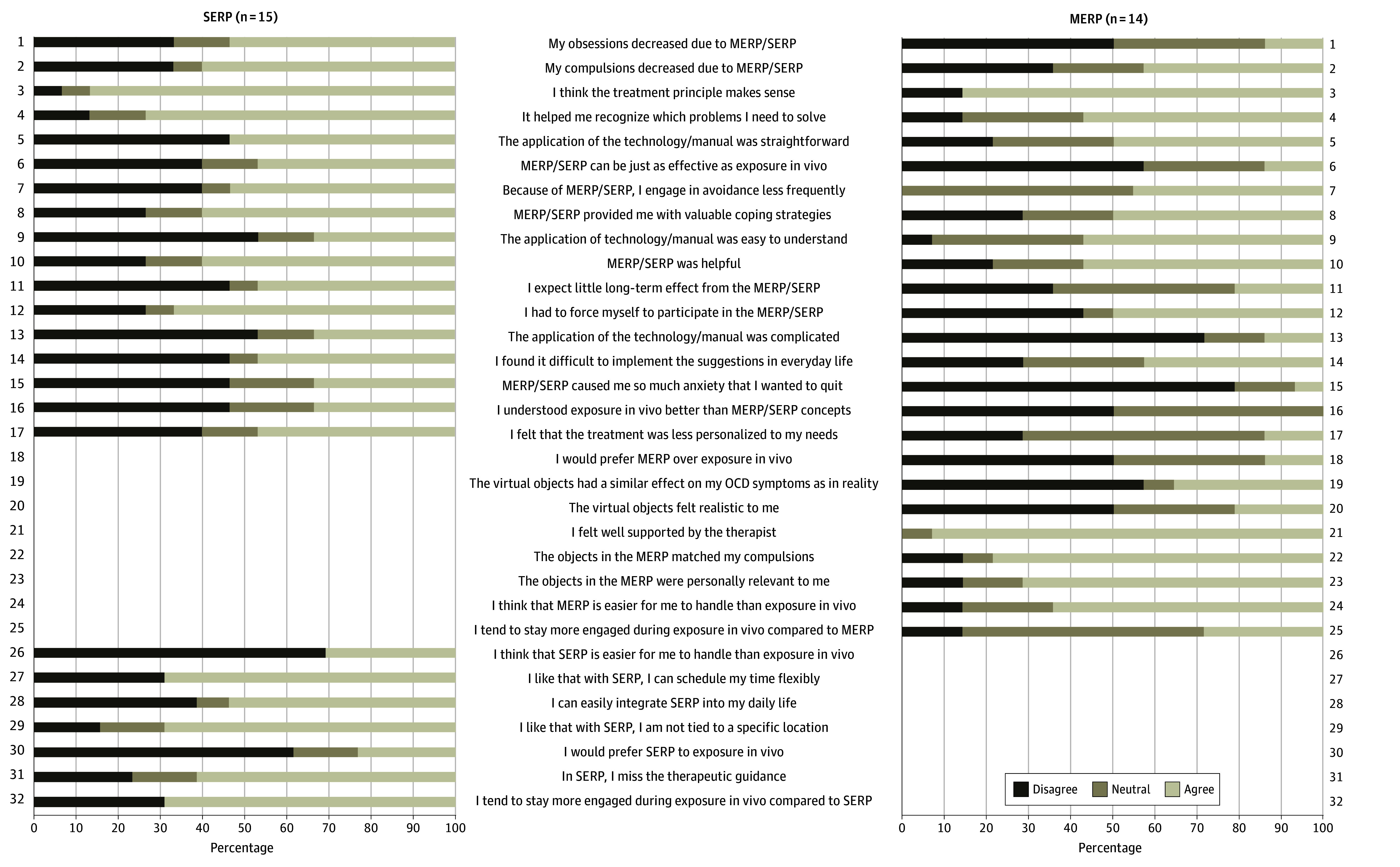
Subjective Appraisal Participants included in the complete-case analysis provided subjective appraisals of self-guided exposure and response prevention (SERP) and mixed-reality exposure and response prevention (MERP). Some items were answered only by the SERP group or only by the MERP group.

The subjective evaluation of MERP and SERP was moderate overall (Figure 3). The 2 groups differed significantly only on the item “I expect little long-term effects from the MERP/SERP,” with a more positive evaluation in the MERP group (χ^2^_4_ = 10.951 [*P* = .03]). In response to the open question about MERP’s advantages compared with in vivo exposure, most patients noted that MERP offers a valuable opportunity for those hesitant to engage in ERP or serves as a first step before in vivo therapy. The primary drawback identified was the constant awareness that the scenario in MERP was not real, leading to a perception of no genuine risk (eg, of contamination). When asked about objects needed for more effective OCD treatment, patients suggested automated teller machine (ATM) keypads, shopping carts, used cleaning cloths, mold, light switches, trash cans, shoes, and food.

### Experiences With Treatment at Baseline and Posttreatment Assessment

Significantly more patients in the MERP group (15 [83.3%]) reported having undergone ERP in the past in comparison with the SERP group at baseline (8 [44.4%]) (χ^2^_1_ = 5.900 [*P* = .02]), but there was no difference at posttreatment assessment for individual therapy or for therapist-guided ERP at posttreatment assessment (referring to the last 6 weeks). Therefore, we did not include it as a covariate in our main analyses. About two-thirds of participants in the SERP group (9 [61.1%]) conducted self-guided ERP at posttreatment assessment, compared with 5 (38.9%) in the MERP group, with no statistically significant difference between groups (χ^2^_1_ = 1.551 [*P* = .21]). Patients in the SERP group completed approximately 4 (mean [SD], 4.29 [2.06]) of 6 exercises.

### Exploratory Analyses

Moderation analyses were conducted exploratorily by including all baseline variables as potential moderators for the change in OCD symptoms (Y-BOCS total score difference from baseline to posttreatment assessment) (eTable in Supplement 2). Positive β coefficients indicate that higher values on the moderator led to a greater improvement in OCD symptoms in the MERP vs the SERP group. Individuals in the MERP group, who had high levels of symptoms at baseline (β range, 3.482 [SE, 1.683] to 3.615 [SE, 1.273]; *P* ≤ .05) and were older (β* = *0.248 [SE, 0.119]; *P* = .05), showed greater reductions in OCD symptoms. Patients in the MERP group who did not indicate a panic disorder at baseline (β* = *–3.114 [SE, 1.416]; *P* = .04) showed greater reductions in OCD symptoms.

## Discussion

The present RCT aimed to investigate the efficacy of a highly innovative MERP compared with SERP in patients with C-OCD. Our findings are summarized as follows.

### Change in Symptoms

Contrary to our primary hypothesis, OCD symptoms (measured by Y-BOCS) did not decrease more in the MERP group than in the SERP group from baseline to post treatment or follow-up, contradicting studies supporting technical-assisted ERPs.^9,10,27^ However, medium to large improvements were seen within the MERP group across various psychopathological variables, including OCD symptoms (Y-BOCS), anxiety (7-item Generalized Anxiety Disorder), and depression (Beck Depression Inventory-II), suggesting potential for improvement with intervention enhancements and more time. Still, only 2 patients in the MERP group (vs 1 in the SERP group) responded by post treatment or follow-up, highlighting limitations in the MERP intervention. Several factors may have influenced the nonsignificant between-group results.

First, the small sample size likely hindered the detection of significant between-group effects. The target was 64 participants, but recruitment challenges, such as limiting participation to patients with C-OCD in our region and COVID-19 restrictions, prevented this. Additionally, the SERP control condition may have been too strong, and the intervention duration too short, necessitating a larger sample to detect significant effects. Future studies should include multiple OCD domains and aim for a larger sample size. Second, the sense of presence in the MERP group was below the midpoint of the scale, reflecting limited engagement with the virtually supplemented environment. This suboptimal sense of presence may have affected efficacy. Previous research on VR-based treatments has shown that a stronger sense of presence is associated with greater emotional engagement,^28^ which in turn improves therapeutic outcomes. Thus, enhancements are needed in future studies to evoke appropriate negative emotions. Third, the ERP sessions may have been too brief and insufficiently intensive, with only 1 session per week for 6 weeks and just 4 exposure sessions. Guidelines recommend exposures on consecutive days and at least 2 lengthy sessions per week,^2^ suggesting that nonsignificant effects may relate more to the delivery format than to technical augmentation. Future studies should explore whether this intensive ERP approach is feasible in MR. Forth, many participants needed 1 to 2 sessions to become familiar with the technology, which may have further reduced the available therapeutic time for effective ERP. This initial adjustment period could have limited MERP’s impact, as less time remained to induce meaningful symptom changes. Last, the significant difference in prior ERP experience between the MERP (83.3%) and SERP groups (44.4%) may have contributed to the lack of significant effects. C-OCD among patients in the MERP group might have been more treatment resistant, which may have limited the patients’ responsiveness to the intervention. Although randomization usually aims to address this, the small sample size may have compromised its effectiveness in balancing these differences.

### Treatment Expectations

Patients’ expectations of MERP were mixed. While all participants anticipated attending all sessions, only 47.2% expected that MERP would help them apply what they learned to everyday life. This skepticism regarding the feasibility of MERP may have been an additional inhibiting factor for recruitment. About half had similar expectations for MERP as for in vivo ERP, indicating aligned anticipated efficacy. Concerns such as dizziness were minimal (<15%), suggesting MR technology fears were not significant barriers. Expectations for OCD symptom improvements were high, with 83.3% expressing optimism. Notably, 94.4% found it easier to confront their OCD in the MR environment than in real life, suggesting MR could facilitate engagement in subsequent in vivo ERP.

### Subjective Appraisal

In evaluating MERP and SERP, subjective ratings were mostly moderate, with a significant difference favoring MERP in long-term benefit expectations. However, few participants preferred MERP over traditional in vivo ERP. Participants were satisfied with the virtual objects used in MERP, finding them relevant and effective for their compulsions. Many viewed MERP as a safer initial step for those hesitant about in vivo ERP or as a precursor to it, which needs to be explored a priori in future studies with a design comparing MERP before ERP vs ERP alone. However, its artificial nature was a common drawback, limiting its impact. Participants also suggested incorporating other objects, such as ATM keypads and shopping carts, to enhance MERP’s therapeutic value and better address individual OCD concerns. The list of stimuli points toward more everyday objects and scenarios that might feel more ecologically valid.

### Exploratory Findings

The moderation analyses sought to identify factors influencing the effectiveness of MERP compared with SERP. Higher baseline symptom severity (Obsessive Compulsive Inventory scores) and older age were associated with more favorable outcomes in MERP, while the absence of panic disorder was also associated with better outcomes. Current research on these moderators is heterogeneous; some studies indicate that high symptom severity leads to poor outcomes,^29^ while others align with our findings.^30^ There is limited research on age as a moderator in technology-assisted interventions, but one study found older adults reported a greater sense of presence in virtual environments, suggesting VR may benefit them more.^31^ The nature of these moderators likely depends on the specific therapy used, indicating a need for more research. Our findings suggest that certain baseline characteristics may interact with treatment type (MERP vs SERP) to influence OCD outcomes, highlighting the importance of individualized treatment approaches and the need for clinicians to consider potential moderators when selecting therapeutic interventions.

### Limitations

Several limitations of this study should be acknowledged. First, the relatively small sample size may limit between-group significance and generalizability due to recruitment challenges tied to a single symptom domain and regional constraints. Future MR devices could allow therapists to appear as avatars, enabling MERP implementation across different locations and increasing geographic flexibility. Technology-supported remote ERP is a promising approach, with options ranging from self-guided exposure exercises at home to more advanced setups where therapists provide virtual guidance via MR headsets. These technological advancements could help bridge treatment gaps and improve access to evidence-based interventions, particularly for patients without direct access to in-person ERP. Second, the focus on patients with C-OCD restricts the applicability of findings to this specific domain; this choice was made to assess MERP’s feasibility and safety. Future studies should include patients with other symptom domains, such as aggressive obsessions, as some therapists may feel uneasy about placing a knife in front of a patient who experiences intrusive thoughts about harming others, to broaden the research scope. Additionally, MERP may not be suitable for all OCD domains, such as symmetry obsessions, where ERP in vivo is typically easy to implement. Third, as a high number of variables was included in the exploratory moderation analyses, the risk of type II error was high. Thus, these results need to be interpreted cautiously. Fourth, the progress of the SERP group was assessed retrospectively, meaning that the actual use may not have been fully captured. Future studies, particularly those offering compensation for participation, should aim to collect real-time data to better track treatment progress. Fifth, a significant proportion of participants in the MERP condition continued concurrent ERP treatment, making it difficult to determine the extent to which the observed effects can be attributed to MERP itself. However, concurrent ERP was also allowed in the control group, ensuring that this factor was at least consistent across conditions. Nevertheless, future studies could address this limitation by implementing the exclusion of concurrent treatments to more clearly isolate the specific effects of MERP. Sixth, the exact dosage of the medications could not be recorded, as patients often did not know or were unsure about it. Therefore, we cannot make any statement regarding the adequacy of the medication. This should be addressed in future studies through physician documentation.

## Conclusions

In this RCT, contrary to our primary hypothesis, OCD symptoms did not significantly decline more in the MERP group compared with the SERP group. However, the MERP group did show some improvements across several psychopathological variables. These findings indicate that while MERP may have potential in addressing OCD and related symptoms, further research is needed to address limitations such as sample size, symptom severity, sense of presence, and session intensity to better assess its efficacy and applicability. Additionally, subjective evaluations suggest that MERP could be considered as a preliminary approach before traditional ERP in vivo.

## Notes

Supplement 1.
Trial Protocol

Supplement 2. **eFigure 1.** Visible Virus Particles on Hand After Touching the Contaminated Object **eFigure 2.** Patients’ Expectations of Mixed Reality Exposure and Response Prevention Therapy (MERP) **eTable.** Moderators for Obsessive-Compulsive Symptom Improvement

Supplement 3.
Data Sharing Statement
