# Supplementary material for: Exposure Therapy in Mixed Reality for Obsessive-Compulsive Disorder: A Randomized Clinical Trial
Source: JAMA Netw Open. 2025 May 20;8(5):e2511488. doi: 10.1001/jamanetworkopen.2025.11488 (PMC12093188; doi:10.1001/jamanetworkopen.2025.11488)
Supplement: Supplement 1. — Trial Protocol [file jamanetwopen-e2511488-s001.pdf]

Trial protocol as submitted to the local ethics committee

Background: Exposure and response prevention therapy (ERP) is an effective treatment with the highest level of evidence for the treatment of anxiety and obsessive-compulsive disorders (OCD) in both international and national guidelines (e.g., DGPPN). Studies confirm the high efficacy of ERP for treating patients with OCD (Abramowitz, Deacon, & Whiteside, 2019). However, it is underutilized in practice due to various reasons (e.g., patients' fear of confronting feared situations or therapists' fear of acute psychological reactions from patients). Research shows that less than 50% of therapists perform ERP with their OCD patients (Külz et al., 2010), and 18.7% of patients discontinue ERP (Ong, Clyde, Bluett, Levin & Twohig, 2016). Thus, there is a significant gap between treatment recommendations and clinical practice, which we aim to address by introducing ERP through Mixed Reality (MR). By implementing ERP in MR, we hope to overcome key treatment barriers. One advantage is that patients would not need to face their fears in real life at first, potentially reducing the fear of treatment. Furthermore, ERP in MR (MERP) could be conducted directly in the therapist's office, reducing time and effort (e.g., travel). In the case of specific phobias, a similar method—Virtual Reality—has been shown to be effective with a large effect size (Carl et al., 2019). Additionally, prior studies on OCD patients have demonstrated that virtual environments can induce the necessary level of anxiety (Belloch et al., 2014; Laforest, Bouchard, Bossé, & Mesly, 2016). MR offers an advantage over VR because it feels more real, as patients can see their real surroundings and therapist while virtual objects are simply added to the environment. This allows for a better addressal of the idiosyncratic nature of OCD.

Objective: The overarching goal of the project is to reduce the treatment gap for patients with OCD by applying MERP and to provide faster assistance to more people. A treatment approach that addresses both the fears and barriers faced by therapists and patients could promise greater success. Barriers to implementation could be significantly reduced by shifting relevant stimuli to a virtual setting.

Approach: A randomized controlled trial will examine the reduction of OCD symptoms through MERP. N = 64 participants will be recruited over 1.5 years. Sample size calculation using G*Power, based on a large effect size (f = .40), power of .80, and alpha = .05, yielded a total sample size of 64. The sample will be divided into an intervention group (participating in MERP) and a control group (participating in self-guided ERP; SERP). Assessments will take place at baseline (t0), after 6 sessions (t1), and after 3 more months (t2). OCD symptomatology will be measured using the gold-standard Yale-Brown Obsessive Compulsive Scale (Y-BOCS) at all three time points. Additional psychopathological symptoms (e.g., depression, BDI-II) and intervention evaluation (e.g., subjective appraisal based on Jelinek et al., 2018) will also be measured. Inclusion criteria include a diagnosis of OCD with contamination-related OCD, a desire for treatment, no acute suicidality, no psychotic or bipolar disorder (current or past), no inpatient treatment (other outpatient treatments may continue), and sufficient knowledge of the German language. The MERP consists of six weekly sessions. The first two sessions focus on assessment and preparation for ERP, and the third session begins the MERP. Participants wear the MR headset, and study therapists use a virtual menu to select OCD-relevant virtual objects (e.g., syringes, bloodstains, used tissues) to place in the therapy room. A guided ERP follows, where participants must interact with the virtual objects. The software will first be tested in a

pilot study with N = 10 inpatients, allowing for important implications for the proposed project. Participants for the pilot study will be recruited from the anxiety and OCD ward at our hospital.

Expected Benefits: In the long term, MERP is expected to be established in clinical settings, leading to faster therapy initiation, preventing inpatient stays, and significantly reducing healthcare costs. This approach could overcome hesitations surrounding ERP and ensure its guideline-compliant and reimbursable application.

More information on the detailed statistical analyses plan can be obtained from Lohse et al. (2023) – a published study protocol: https://link.springer.com/article/10.1186/s40359-023-01116-3
