# Supplementary material for: Exposure Therapy in Mixed Reality for Obsessive-Compulsive Disorder: A Randomized Clinical Trial
Source: JAMA Netw Open. 2025 May 20;8(5):e2511488. doi: 10.1001/jamanetworkopen.2025.11488 (PMC12093188; doi:10.1001/jamanetworkopen.2025.11488)
Supplement: Supplement 2. — eFigure 1. Visible Virus Particles on Hand After Touching the Contaminated Object eFigure 2. Patients’ Expectations of Mixed Reality Exposure and Response Prevention Therapy (MERP) eTable. Moderators for Obsessive-Compulsive Symptom Improvement [file jamanetwopen-e2511488-s002.pdf]

Supplementary Online Content

Miegel F, Jelinek L, Lohse L, et al. Exposure therapy in mixed reality for obsessive-compulsive disorder: a randomized clinical trial. *JAMA Netw Open.* 2025;8(5):e2511488. doi:10.1001/jamanetworkopen.2025.11488

eFigure 1. Visible Virus Particles on Hand After Touching the Contaminated Object

eFigure 2. Patients' Expectations of Mixed Reality Exposure and Response Prevention Therapy (MERP)

eTable. Moderators for Obsessive-Compulsive Symptom Improvement

This supplementary material has been provided by the authors to give readers additional information about their work.

© 2025 Miegel F et al. *JAMA Network Open*.

eFigure. Visible Virus Particles on Hand After Touching the Contaminated Object

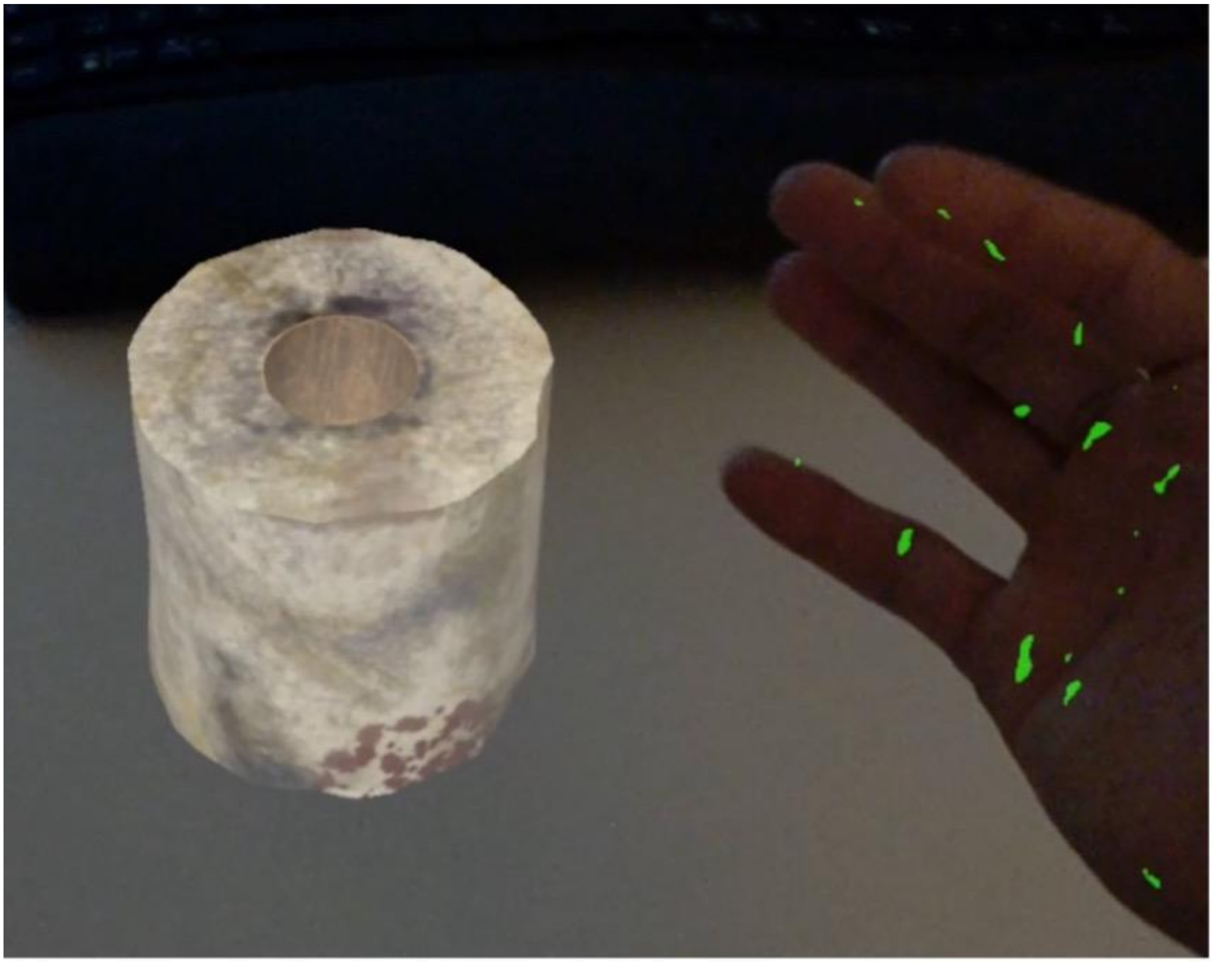

© 2025 Miegel F et al. *JAMA Network Open*.

eFigure 2. Patients' Expectations of Mixed Reality Exposure and Response Prevention Therapy (MERP)

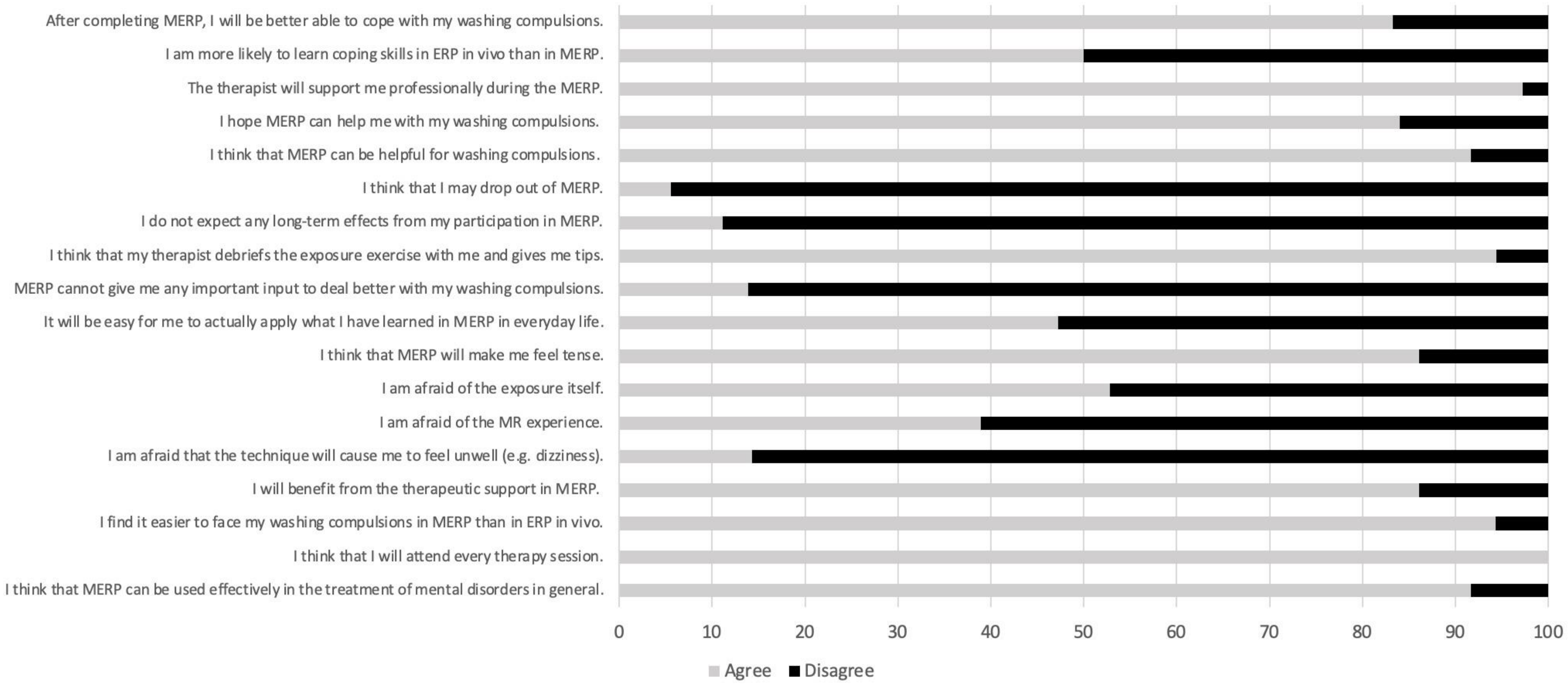

© 2025 Miegel F et al. *JAMA Network Open*.

Supplementary Material C

eTable. Moderators for Obsessive-Compulsive Symptom Improvement

(Y-BOCS Total Difference Scores; Means Are Centered).

Outcome Parameter	*B*	*SE*	*t*	*p*	LLCI	ULCI	*p* for –1 SD	0	*p* for +1 SD
Age	0.248	0.119	2.095	.05	0.005	0.491	.42	.617	.04
Panic disorder	–3.114	1.416	–2.200	.04	–6.020	–0.209	.10	.098	.23
Thought control (OCI-R item 6; T0)	3.568	1.232	2.896	.01	1.040	6.095	.04	.071	.07
Repetition of numbers (OCI-R item 10; T0)	3.482	1.683	2.069	.05	0.029	6.934	.67	.668	.15
Checking of faucet and light (OCI-R item 14; T0)	3.615	1.273	2.839	.01	1.002	6.227	.23	.540	.01

Notes. OCI-R = Obsessive Compulsive Inventory-Revised; B = beta coefficient, SE = standard error, LLCI = lower limit confidence interval, ULCI = upper limit confidence interval. The last three columns present the *p*-values when the values are one standard deviation below, above, and equal to the mean.

© 2025 Miegel F et al. *JAMA Network Open*.
