# Supplementary material for: Exposure Therapy in Mixed Reality for Obsessive-Compulsive Disorder: A Randomized Clinical Trial
Source: JAMA Netw Open. 2025 May 20;8(5):e2511488. doi: 10.1001/jamanetworkopen.2025.11488 (PMC12093188; doi:10.1001/jamanetworkopen.2025.11488)
Supplement: Supplement 3. — Data Sharing Statement [file jamanetwopen-e2511488-s003.pdf]

Data Sharing Statement

Miegel. Exposure Therapy in Mixed Reality for Obsessive-Compulsive Disorder. *JAMA Netw Open*. Published May 20, 2025. doi:10.1001/jamanetworkopen.2025.11488

Data
Additional Information: German Registry of Clinical Trials: DRKS00020969 URL: https://www.bfarm.de/DE/Das-BfArM/Aufgaben/Deutsches-Register-Klinischer-Studien/_node.html
Data available: Yes
Data types: Deidentified participant data
How to access data: The data supporting the results and analyses presented in this paper are available upon request from the first author.
When available: With publication

Supporting Documents
Document types: None

Additional Information
Who can access the data: To anyone requesting the data.
Types of analyses: Meta-analyses
Mechanisms of data availability: With investigator support
